# Cytoprotective mechanism of ferulic acid against high glucose-induced oxidative stress in cardiomyocytes and hepatocytes

**DOI:** 10.3402/fnr.v60.30323

**Published:** 2016-02-10

**Authors:** Yuan Song, Luona Wen, Jianxia Sun, Weibin Bai, Rui Jiao, Yunfeng Hu, Xichun Peng, Yong He, Shiyi Ou

**Affiliations:** 1 Out-patient Department, First Affiliated Hospital, Jinan University, Guangzhou, China; 2 Department of Food Science and Engineering, Jinan University, Guangzhou, China; 3 Faculty of Chemical Engineering and Light Industry, Guangdong University of Technology, Guangzhou, China

**Keywords:** ferulic acid, oxidative stress, cardiomyocytes, hepatocytes

## Abstract

**Background:**

Ferulic acid (FA), a phenolic acid, is a potential therapy for diabetes mellitus. FA has been shown to protect against hepatic and myocardial injury and oxidative stress in obese rats with late-stage diabetes, but the mechanism of the antioxidative activity of FA is still unclear.

**Objective:**

The aim of this study was to elucidate whether FA can prevent damage to cardiomyocytes and hepatocytes caused by high glucose (HG)-induced oxidative stress and whether the protection effects of FA on these cells are related to the Keap1-Nrf2-ARE signaling pathways.

**Design:**

Cells were divided into four groups: a control group (cultured with normal medium), an HG group (medium containing 80 mmol/L glucose), an FA+HG group (medium containing 80 mmol/L glucose and 1, 5, or 10 µg/mL FA), and a dimethylbiguanide (DMBG)+HG group (medium containing 80 mmol/L glucose and 50 µg/mL DMBG).

**Results:**

FA treatment significantly increased cell viability and significantly decreased cell apoptosis compared with the HG-treated group. Moreover, FA down-regulated the expression of Keap1 protein and up-regulated the expression of Nrf2 protein and gene transcription of HO-1 and glutathione S-transferase (GST) in a dose-dependent manner.

**Conclusion:**

FA alleviated the HG-induced oxidative stress and decreased cell apoptosis in hepatocytes and cardiomyocytes. These effects were associated with the Keap1-Nrf2-ARE signaling pathway.

Ferulic acid (FA), a phenolic acid, is a component of Chinese medicinal herbs such as *Ligusticum chuangxiong*, *Angelica sinensis*, and *Cimicifuga racemosa* (1). FA is also abundant in fruits, vegetables, and some beverages including beer and coffee (2, 3) and has been proposed as a potential agent for diabetes mellitus (DM), cancer, Alzheimer's disease, skin disease, and cardiovascular diseases (4–6). In a DM animal model, FA elicited beneficial effects by scavenging free radicals and activating antioxidant enzymes, resulting in a reduction in the cellular redox imbalance (7–9). In a *db/db* mouse model, oral administration of FA for 17 days was reported to reduce blood glucose and increase plasma insulin (10). In addition, the administration of FA for 3 weeks led to blood glucose reduction in streptozotocin-treated diabetic rats (11).

DM is a chronic disorder characterized by an increased concentration of glucose in the blood. Hyperglycemia, resulting from uncontrolled glucose regulation, can induce overproduction of ROS, mainly the superoxide anion, by the mitochondrial electron-transport chain (12). Oxidative stress plays a significant role in diabetes and diabetes complications because it induces the overproduction of free radicals and impairs antioxidant defenses (13). Thus antioxidants, especially polyphenols, may be good supplements for diabetes management (14, 15).

FA is a potential therapy for DM due to its efficiency as an antioxidant with few side effects, but the mechanism of the antioxidative activity of FA is still unclear. In a previous study, we showed that FA protects against hepatic and myocardial injury and oxidative stress in obese rats with late-stage diabetes (16, 17). It was reported that Keap1-Nrf2-ARE played a key role in the protection against oxidative stress and the toxicity of exogenous substances (18, 19). In the present study, primary rat cardiomyocytes and hepatocytes cultured *in vitro* were used to determine whether the Keap1-Nrf2-ARE signaling pathway was involved in the mechanism of cytoprotection induced by FA.

## Materials and methods

### Primary rat hepatocyte isolation and culture

In this study, 1- to 3-day-old neonatal Sprague–Dawley male rats [NO4405900280, permitted by SCXK 2008-0020 (Guangdong)] were purchased from the Experimental Animal Center of Guangdong Province, China, and were used as hepatocyte and cardiomyocyte donors. Briefly, livers isolated from the rats were washed in Hank's solution at 4°C and minced. Liver tissue blocks were washed with ice-cold Hank's solution three times and were then incubated with Hank's solution supplemented with 5 mmol/L CaCl_2_, 0.3 g/L collagenase (Type II; Sigma, Munich, Germany) and 0.05 g/L DNase (Sigma) at 37°C for 15 min. Next, the supernatant was collected and mixed with Hank's solution containing 5% fetal bovine serum (FBS; Hyclone, Logan, Utah, USA) and 45% Roswell Park Memorial Institute (RPMI) 1640 medium (Hyclone) at 4°C. The incubation and collection procedures were repeated twice, and the collected supernatant was passed through a stainless steel sieve (200 meshes per inch) and then centrifuged at 400 g for 10 min. After centrifugation, the precipitate was resuspended in 10 mL of 70% Percoll solution (Sigma) and gently placed on the surface of 20 mL of 30% Percoll solution in a 50-mL centrifuge tube. Then, 20 mL of Dulbecco's modified Eagle's medium (DMEM; Hyclone) was added to the surface of the cell suspension. The mixture was centrifuged for 30 min at 800 g, and the suspension located between the scale marks 10 mL and 25 mL on the tube was collected. The collected suspension was mixed with 20 mL RPMI 1640 medium supplemented with 10% FBS and was then centrifuged at 800 g for 10 min. The precipitate was resuspended with RPMI 1640 medium containing 80 mmol/L glucose and supplemented with 8% FBS.

The hepatocytes were counted using a hemocytometer, and hepatocyte viability was assessed by trypan blue staining, regarding stained cells as dead.

### Primary rat cardiomyocyte isolation and culture

Hearts isolated from the rats were washed in phosphate buffer saline (PBS), minced, and digested with 0.25% trypsin at 4°C overnight. FBS was then added to the solution to stop the digestion. The mixture was centrifuged at 1,000 r/min for 5 min, and the supernatant was discarded. The precipitate was resuspended with 0.8% collagenase type II and agitated with a magnetic stirrer for 15 min. The supernatant was collected and again digested with 0.8% collagenase type II. The digestion steps were repeated several times until no visible tissue blocks existed. Then the mixture was centrifuged, resuspended with HG DMEM medium containing 80 mmol/L glucose, supplemented with 8% FBS, and placed in culture flasks in a 5% CO_2_ atmosphere at 37°C. The first and second times the medium was replaced, 5-Bromo-2-deoxy uridine (Sigma) was added to the medium to deplete the non-myocytes.

### Immunofluorescence

Primary rat hepatocytes and cardiomyocytes isolated from rats were cultured as described above on polylysine-coated coverslips. Cells were fixed with ice-cold acetone for 15 min and blocked for 30 min with 1% bovine serum albumin (BSA; Sigma) in PBS. Then the cells were incubated for 2 h at 37°C with primary monoclonal antibodies diluted at 1:1,000 in blocking solution. After being washed three times in excess PBS, the cells were incubated for 1 h at 37°C with fluorescein isothiocyanate (FITC)-labeled anti-rabbit IgG antibody (Life Technologies, Carlsbad, CA, USA) diluted at 1:100 in blocking solution. After been rinsed with PBS, the samples were analyzed by fluorescent microscopy (Leica, Nussloch, Germany). Rabbit anti-rat CK-18 antibody (Abcam, Cambridge, UK) was used asthe primary monoclonal antibody to identify hepatocytes, and rabbit anti-rat actin antibody (Abcam) was used to identify cardiomyocytes.

### Experimental groups and drug treatment

Cells were divided into four treatment groups: 1) a control group: cells cultured with normal medium (the DMEM contained 25 mmol/L glucose and the RPMI 1640 medium contained 11 mmol/L glucose); 2) a high glucose (HG)-treated group: cells cultured with medium containing total 80 mmol/L glucose; 3) FA- and HG-treated groups: cells cultured with medium containing 80 mmol/L glucose and supplemented with 1, 5, or 10 µg/mL FA (purity>99%, Aladdin, Shanghai, China); and 4) a dimethylbiguanide- (DMBG) and HG-treated group: cells cultured with medium containing 80 mmol/L glucose and with a supplementation of 50 µg/mL DMBG (Bristol-Myers Squibb, Shanghai, China). The final glucose concentration up to 80 mmol/L was obtained by diluting D-glucose solution (Sigma) with DMEM or RPMI 1640 medium.

### Cell viability

Cells were seeded at a density of 10,000 cells per well in 96-well plates and cultured in an atmosphere of 5% CO_2_ at 37°C in the media described above. After 48 h of culture, 20 µL of MTT (Sigma; 5 mg/mL in PBS) was added to each well, and the cells were incubated at 37°C for 4 h. Next, the supernatant was replaced by 150 µL DMSO per well to dissolve blue formazan crystals, and the absorbance was measured at 570 nm using an MK3 microplate reader (Thermo, Waltham, MA, USA).

### Apoptosis assay

Cell apoptosis was detected by flow cytometry using the Annexin V-FITC apoptosis detection kit (Life Technologies). Briefly, cells were seeded in 6-well plates at a density of 100,000 cells per well. After being cultured for 24 h, the cells were detached with 0.25% trypsin without ethylenediaminetetraacetic acid and were resuspended in binding buffer at a density of 1×10^6^ cells/mL. Subsequently, 5 µL of Annexin V solution and 1 µl of PI solution were added to 100 µL of the cell suspensions in tubes. After incubation for 15 min at ambient temperature, 400 µL of binding buffer was added to the tubes while they were on ice. Flow cytometry analysis was then performed using a BD FACScalibur flow cytometer (BD Biosciences, Heidelberg, BW, Germany).

### RNA isolation and real-time Polymerase Chain Reaction (PCR)

Total RNA was extracted from cells using Trizol reagent (Life Technologies) according to the manufacturer's instructions. The quality of the RNA was assessed by measuring the A_260_/A_280_ ratio with a microplate reader. First-strand cDNA was synthesized using a first-strand cDNA Synthesis Kit (Tiangen Biotech Co., Ltd., Beijing, China), and Polymerase Chain Reaction (PCR) was carried out with a Real Master Mix (SYBR Green) kit (Tiangen Biotech Co., Ltd.) using a CFX96 System (Millipore, Billerica, MA, USA). The comparative C_T_ method was used to quantify the expression of the gene being studied using reduced glyceraldehyde-phosphate dehydrogenase (GAPDH) as the normalization control. The primers were as follows: HO-1, forward (5′→3′) TGCACATCCGTGCAGAGAAT and reverse (5′→3′) CTGGGTTCTGCTTGTTTCGC; GST, forward (5′→3′) CTAGCTGTCCTCCTGGGATTC and reverse (5′→3′) CTAGCTGTCCTCCTGGGATTC; GAPDH, forward (5′→3′) CTCAGTTGCTGAGGAGTCCC and reverse (5′→3′) ATTCGAGAGAAGGGAGGGCT.

### Western blot

For protein isolation, cells were lysed using radio immunoprecipitation assay (RIPA) lysis buffer (BioTeke, Beijing, China). Nuclear and cytoplasmic protein fractions were extracted separately to quantify the concentration of Nrf2 in the nucleus and the concentration of Keap1 in the cytoplasm. Protein concentration was determined using a bicinchoninic acid (BCA) assay kit (Shennengbocai Biotech, Shanghai, China). The samples were electrophoresed in a 10% sodium dodecyl sulfonate-polyacrylamide gel at 120 V and were transferred to polyvinylidene difluoride membranes (Millipore, Billerica, MA, USA). The membranes were blocked at room temperature for 2 h with BSA and incubated at 4°C overnight using relative primary antibodies at 1:1,000: rabbit anti-rat Keap1 antibody (CST, Boston, MA, USA), rabbit anti-rat Nrf2 antibody (Abcam), rabbit anti-rat GAPDH antibody (CST), and rabbit anti-rat histone H3 antibody (CST). The proteins were then incubated with horseradish peroxidase-conjugated secondary antibody (Jackson ImmunoResearch, West Grove, PA, USA) diluted at 1:2,000 in tris-buffered saline with Tween for 1 h at room temperature. The specific signals were detected by enhanced chemiluminescence (Thermo) following the manufacturer's protocol.

### Statistical analysis

The experiments were performed three times. Data are presented as the means±standard error of the mean, and different groups were analyzed by one-way analysis of variance. *P<*0.05 was considered to be statistically significant.

## Results

### Hepatocytes and cardiomyocytes in vitro

**Fig. 1:**
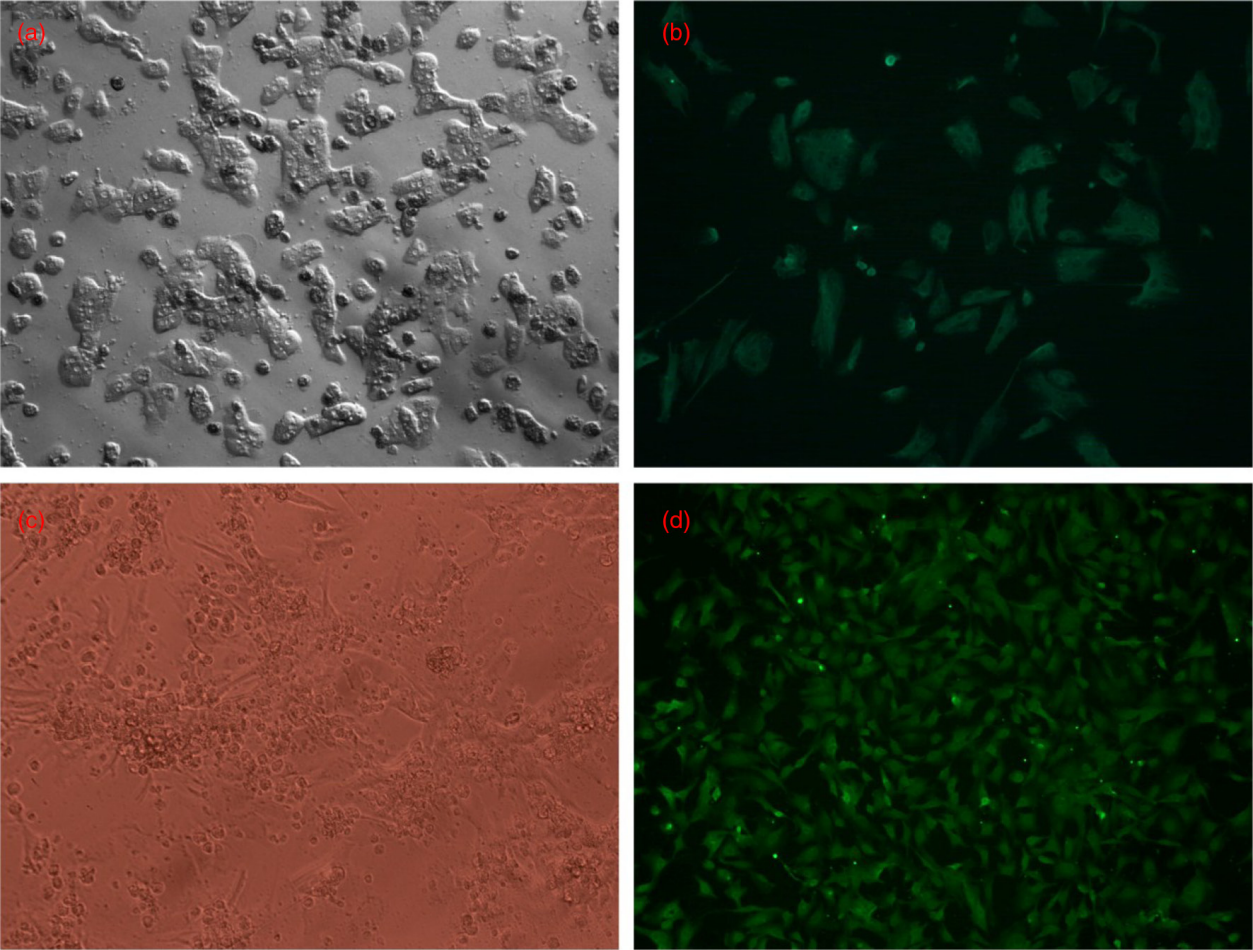
Hepatocytes and cardiomyocytes *in vitro*. (a) Hepatocytes in culture (200×). (b) CK-18 (green) staining of hepatocytes (200×). (c) Cardiomyocytes in culture (100×). (d) Sarcomeric actin (green) staining of cardiomyocytes (100×).

The trypan blue staining assessment showed that 95% of the isolated rat hepatocytes were viable *in vitro*. According to the immune fluorescence analysis, over 90% of hepatocytes expressed CK-18 (Fig. 1a and b) and 90–95% of cardiomyocytes expressed actin (Fig. 1c and d). These results indicate that the isolated hepatocytes and cardiomyocytes were of high purity.

### Effect of FA on cell viability

**Fig. 2:**
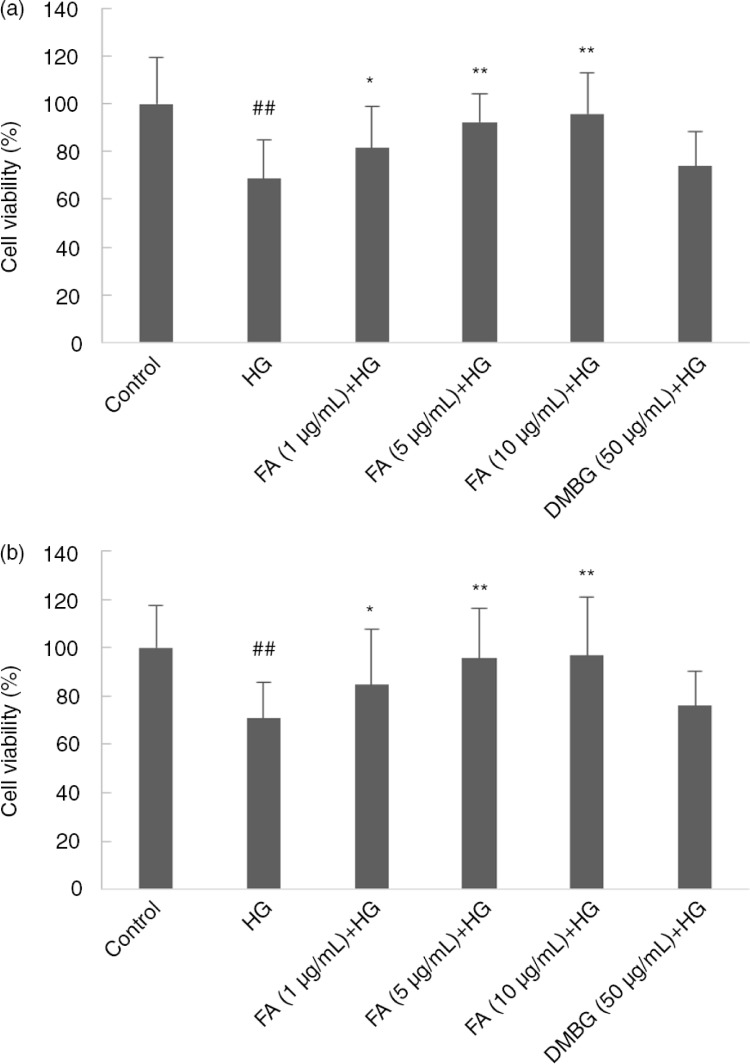
Effect of ferulic acid (FA) and dimethylbiguanide (DMBG) on (a) hepatocyte and (b) cardiomyocyte viability. Mean±SEM, *n*=3. ^##^ *p*<0.01 vs. control. *p<0.05 vs. high glucose-treated group. **p<0.01 vs. high glucose-treated group.

The cell viability of the model group was significantly lower than that of the control group (*p*<0.01). The FA-treated group showed a dose-dependent increase in cell viability compared with the control (*p<*0.05, *p<*0.01). Thus 1, 5, and 10 µg/mL FA protected hepatocytes and cardiomyocytes against cell death induced by HG. DMBG did not significantly increase cell viability (*p>*0.05) (Fig. 2).

### Effect of FA on cell apoptosis

**Fig. 3:**
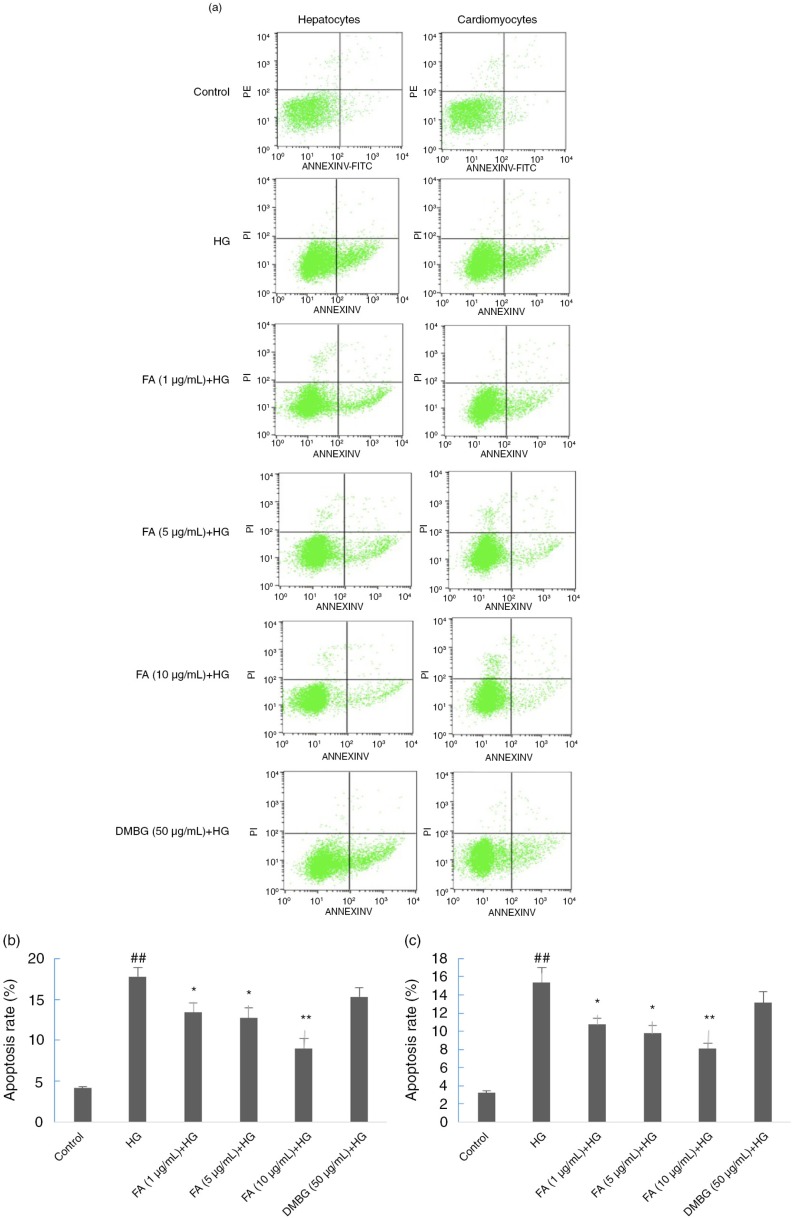
Effects of ferulic acid (FA) and dimethylbiguanide (DMBG) on apoptosis of hepatocytes and cardiomyocytes. (a) Flow cytometry results. (b) Apoptotic rate of hepatocytes. (c) Apoptotic rate of cardiomyocytes. Mean±SEM, *n*=3. ^##^ *p<*0.01 vs. control. *p<0.05 vs. high glucose-treated group. **p<0.01 vs. high glucose-treated group.

The apoptotic rates of the hepatocytes and cardiomyocytes of the HG-treated group increased significantly compared with those of the control group. Compared with the HG-treated group, in the FA-treated group the proportion of early and late apoptotic cells was reduced with increasing FA concentration (*p<*0.01). In the DMBG-treated group, the proportion of normal and apoptotic cells was similar to that of the HG-treated group (*p>*0.05). Thus, FA protected hepatocytes and cardiomyocytes against apoptosis induced by HG, whereas DMBG did not significantly reduce cell apoptosis (Fig. 3).

### Effect of FA on transcription of HO-1 and GST

**Fig. 4:**
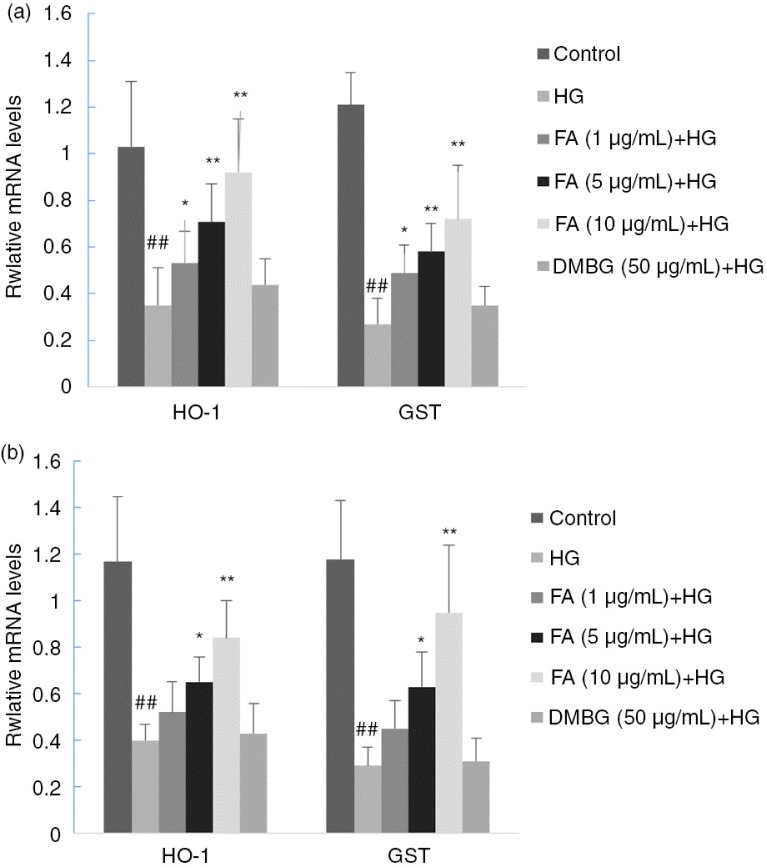
Effects of ferulic acid (FA) and dimethylbiguanide (DMBG) on gene transcription levels of HO-1 and GST in (a) hepatocytes and (b) cardiomyocytes. Mean±SEM, *n*=3. ^##^ *p<*0.01 vs. control. *p<0.05 vs. high glucose-treated group. **p<0.01 vs. high glucose-treated group.

HO-1 and GST are two important enzymes responsible for protection against oxidative stress. HO-1 is a rate-limiting enzyme that catalyzes the degradation of heme and produced iron, carbon monoxide, biliverdin, and enzymatic products, which exert antioxidative functions through various mechanisms (20). GST plays an important role in the cellular defense system and helps cells respond to oxidative stress (21). The transcription of HO-1 and GST genes was found to be decreased in the presence of a high concentration of glucose and increased with increasing concentrations of FA. DMBG did not significantly affect the mRNA levels of HO-1 or GST (*p>*0.05) (Fig. 4).

### Effect of FA on protein expression of Nrf2 and Keap1

**Fig. 5:**
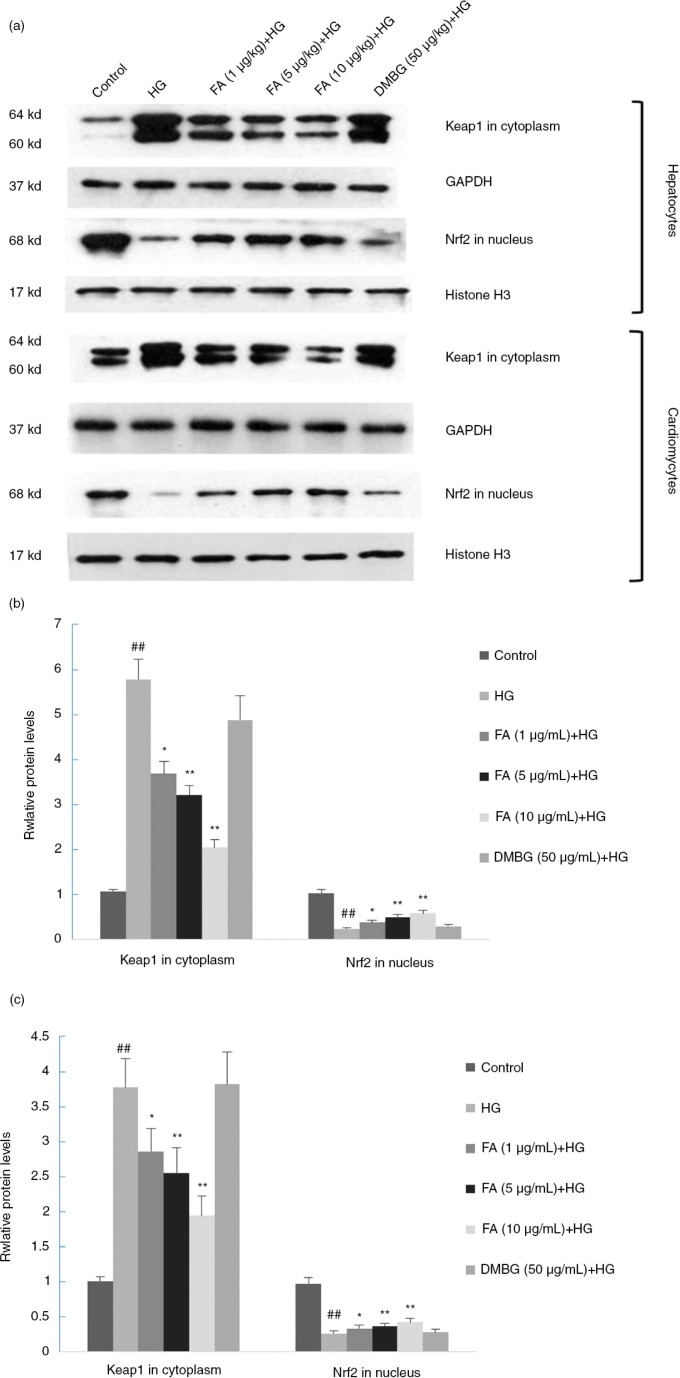
Effects of ferulic acid (FA) and dimethylbiguanide (DMBG) on Keap-1 and Nrf2 expression in hepatocytes and cardiomyocytes. (a) Western blot results. (b) Keap-1 and Nrf2 expression levels in hepatocytes. (c) Keap-1 and Nrf2 expression levels in cardiomyocytes. Mean±SEM, *n*=3. ^##^ *p<*0.01 vs. control. *p<0.05 vs. high glucose-treated group. **p<0.01 vs. high glucose-treated group.

In the HG-treated group, the concentration of Nrf2 was lower and the concentration of Keap1 was higher than those of the control group (*p<*0.05). FA promoted Keap1 expression in the cytoplasm, where Keap1 could degrade and release Nrf2 (*p<*0.05). DMBG did not have a significant effect on the protein expression of Nrf2 or Keap1 (*p>*0.05) (Fig. 5).

## Discussion

Hyperglycemia is the hallmark sign of diabetes as well as one of the causes of diabetes complications. For the past few years, uncontrolled regulation of blood glucose-induced injury and complications in the heart and liver have drawn considerable attention. Brownlee proposed a simple hyperglycemia-induced process, overproduction of superoxide by the mitochondrial electron-transport chain, which generated diabetic complications (22).

FA and FA ethyl esters have been shown to enhance cell stress response by regulating several critical enzymes, such as HO-1, GST, quinine oxidoreductase, glutamate-cysteine ligase, catalase, and superoxide dismutase. In preclinical models, including gerbil synaptosomes, rat neurons, and dermal fibroblasts, FA and FA ethyl esters were shown to up-regulate HO-1 expression (23–26), which is consistent with the results of the present study. Increased expression of HO-1 had a significant effect on glucose oxidase– and ROS-related oxidative damage. GST down-regulation was also detected in FA-treated preclinical models (27–29).

FA has shown antioxidant activity (30) and is regarded as a potential supplement to manage diabetes (1, 11). In our previous study on the heart and liver of rat models of diabetes, FA induced the up-regulation of the gene expression of HO-1 and GST (*p<*0.05), indicating that FA may protect against oxidative stress in the heart and liver of obese rats with late-stage diabetes (16).

One possible mechanism of FA-induced HO-1 up-regulation is through the Keap1-Nrf2-ARE signaling pathway. Nrf2 is a key regulator of HO-1 that acts by binding to ARE, a *cis*-acting enhancer sequence that mediates the transcriptional activation of Nrf2, in response to oxidative stress (31). Under unstressed conditions, Nrf2 exists as an inactive complex with its repressor Keap1, which negatively regulates Nrf2 by ubiquitination and proteasomal degradation (32, 33). Once stimulated by inducers such as ROS and electrophilic reagents, Nrf2 dissociates from Keap1, translocates into the nucleus, and binds to ARE. These phenomena result in the regulation of target genes, including HO-1 and GST (34, 35).

To confirm the hypothesis that FA was involved in the Keap1-Nrf2-ARE signaling pathway, we designed the cell model used in the present study. Hepatocytes and cardiomyocytes were treated with 80 mmol/L glucose, which resulted in a decrease in cell viability and an increase in apoptosis. Furthermore, Nrf2 protein expression was down-regulated, Keap1 protein expression was up-regulated, and HO-1 and GST transcription was up-regulated significantly. Thus, the injury to hepatocytes and cardiomyocytes induced by HG may be connected to the Keap1-Nrf2-ARE signaling pathway. FA relieved the effects of HG on hepatocytes and cardiomyocytes. Keap1 protein expression in the nucleus was down-regulated in FA-treated groups compared with HG groups. As a result, more Nrf2 molecules dissociated from Keap1 and escaped from proteasomal degradation. Hence, Nrf2 accumulation in the nucleus increased, which further up-regulated the gene expression of antioxidant enzymes such as HO-1 and GST.

It has been reported that silencing of the Nrf2 gene abrogates ellagic acid-induced increases in HO-1 expression in human umbilical venous endothelial cells. This result suggests that ellagic acid, a phenolic compound, protects human umbilical venous endothelial cells against oxidative stress-induced injury by the Keap1-Nrf2-ARE signaling pathway (36). FA, which is also a phenolic acid, may reduce oxidative damage through a mechanism similar to that of ellagic acid.

HG-induced oxidative stress in cardiomyocytes and hepatocytes was examined in the present study. However, advanced glycation end products (AGEs), which play a major role in triggering oxidative damage, could be synthesized in cells exposed to HG (29). In our previous study, the effect of FA on HG-induced oxidative stress in the heart and liver was assessed, and the concentration of AGEs was measured. It was found that AGEs increased in HG-treated groups compared with the control and decreased in FA-treated groups compared with HG-treated groups (16). Therefore, we hypothesize that the oxidative stress in HG-treated cells may be induced by glucose itself or by AGEs; further studies are warranted.

Studies have shown that HG decreases cell viability, increases apoptosis, down-regulates the expression of Nrf2 protein, and up-regulates the expression of Keap1 protein in both cardiomyocytes (31, 37) and hepatocytes (38). Moreover, FA protects against HG-induced oxidative stress through the same signaling pathway in both of the cells. It leads to a similar pattern of figures, such as that shown in Figs. 2a and b, 3b and c, and 5b and c.

DMBG, a commercial oral drug for the treatment of type 2 diabetes, is one of the most commonly prescribed drugs worldwide due to its safety and low cost (39). Cell viability and apoptosis were not significantly affected by DMBG treatment, indicating that DMBG did not show a protection effect against glucose-induced hepatocyte and cardiomyocyte injury. Moreover, in the DMBG-treated group, the protein expression levels of Nrf2 and Keap1 and the transcription levels of GST and HO-1 were not significantly different from those of the HG-treated group, suggesting that the effect of DMBG was not achieved through the Keap1-Nrf2-ARE signaling pathway.

In conclusion, FA improved the antioxidative stress ability of hepatocytes and cardiomyocytes exposed to HG. The mechanism was associated with the Keap1-Nrf2-ARE signaling pathway. The protection effect of DMBG against glucose-induced hepatocyte and cardiomyocyte injury was not significant.
